# Artificial Intelligence-Based Automated Detection of Chest X-ray Abnormalities as Support for Young Radiologists

**DOI:** 10.2174/0115734056407511250904090252

**Published:** 2026-07-30

**Authors:** Luca Giuliani, Giorgio Maria Masci, Nicholas Landini, Leonardo Bosi, Cosimo Nardi, Flaminia De Cristofaro, Stefano Perotti, Valeria Panebianco, Paolo Giuliani, Carlo Catalano

**Affiliations:** 1 Policlinico Umberto I Hospital, Department of Radiological and Oncological Sciences and Pathological Anatomy, Sapienza University, 00161 Rome, Italy; 2 Policlinico Umberto I Hospital, Department of Translational And Precision Medicine, Sapienza University, 00161 Rome, Italy; 3 Department of Experimental and Clinical Biomedical Sciences, Radiodiagnostic Unit n. 2, University of Florence-Azienda OspedalieroUniversitaria Careggi, Largo Brambilla 3, 50134, Florence, Italy; 4 Poliambulatorio Montezemolo, 00195 Rome, Italy

**Keywords:** AI-based software, Chest X-ray, Computer-aided detection, Young Radiologists, Radiological examination, AI-tools

## Abstract

**Introduction:**

Chest X-ray (CXR) still represents the most performed radiological examination, but its interpretation may differ among readers. In the last years, several automatic detection tools have been developed to assist radiologists in CXR interpretation. We aimed to evaluate how artificial intelligence (AI)-based software may increase the performance of young radiologists in CXR interpretation compared to that of an experienced CXR radiologist.

**Materials and Methods:**

500 CXRs were selected to generate a well-balanced dataset. For each patient, the CXR reading was conducted by an AI-based software (Lunit INSIGHT CXR) and, independently, by two general radiologists with less than one year of experience. Furthermore, after three months, the radiologists reviewed all the examinations with the assistance of the software. The following CXR findings were searched: atelectasis, calcification, cardiomegaly, consolidation, fibrosis, nodules, mediastinal widening, pleural effusion, pneumoperitoneum, and pneumothorax. Sensitivity and specificity were computed compared to an expert reader allowed to use AI assistance. Chi-squared tests were used to compare the readings.

**Results:**

A total of 600 findings were identified by the senior radiologist. The sensitivity of young radiologists without AI was significantly lower than that of AI (*p* < 0.001), while the specificity was significantly higher for both (*p* < 0.001). With the assistance of AI, overall sensitivity increased in both radiologists (*p* < 0.001), while specificity decreased (p ≤ 0.004).

**Discussion:**

AI software achieves very high sensitivity, minimizing the number of false negatives as much as possible. As a counterpart, there could be a non-negligible risk of overdiagnosis.

**Conclusion:**

AI-based software assistance can yield a potential enhancement in sensitivity for young radiologists in the interpretation of CXRs. However, there might be a reduction in specificity.

## 1 INTRODUCTION

Chest X-ray (CXR) still represents the most performed radiological examination due to its large availability, low cost, and low radiation dose, remaining a first-line examination in a wide range of conditions [1]. However, the interpretation of CXR is often challenging due to the superimposition of anatomical structures, non-homogeneous image quality, and low-contrast resolution. Hence, radiologists usually show high interobserver variability when interpreting CXR, depending also on the experience of the readers, and lower accuracy compared to Computed Tomography (CT), the reference imaging tool for chest imaging [2].

For instance, compared to CT, CXR without the aid of artificial intelligence (AI) may show a sensitivity ranging from 76% to 82% in the detection of lung cancer.

For these reasons, in recent years, several automatic detection tools have been developed to assist radiologists in CXR interpretation, including systems providing standard computer-aided detection and those developed with deep-learning algorithms [3, 4], showing high sensitivity for the detection of many chest abnormalities, such as consolidation, pleural effusion, lung nodules, and pneumothorax [5-7]. Moreover, the advancements in deep learning techniques have demonstrated remarkable efficacy, allowing software analysis to surpass even the accuracy of human evaluation in some studies [8-10].

Therefore, in this study, we aimed to evaluate how valid the diagnostic performance of AI is in comparison to that of two young radiologists and whether radiological interpretation can achieve a better diagnostic performance with AI assistance for young general radiologists compared to an experienced CXR radiologist.

## 2 MATERIALS AND METHODS 

This study received approval from the local ethics committee (Sapienza University of Rome, Rif. 7226, Prot. 0473/2024). The need for informed consent was waived because of the retrospective nature of the study.

### 2.1 Study Design

All consecutive CXR examinations performed at Poliambulatorio Montezemolo in Rome between March 2023 and January 2024 were retrospectively identified from the picture archiving and communication system (PACS) by a senior radiologist with more than 10 years of experience in thoracic radiology.

The inclusion criteria were the availability of the Postero-Anterior (orthostatic) or Antero-Posterior (supine) projection, while the exclusion criteria were studies with technical faults or incorrect positioning [11] and age under 14 years old, on whom the software had not been tested. Inclusion and exclusion criteria were evaluated in consensus by two radiology residents of the Sapienza University.

The first 500 CXRs fitting the criteria were included.

### 2.2 Artificial Intelligence-Based Software Analysis

Lunit INSIGHT CXR, Version 3.1.1.0, is a computer-aided detection software based on an artificial intelligence algorithm designed for use as a concurrent reading aid. It was developed based on a dataset of 146,717 chest radiographs obtained from Seoul National University Hospital [12].

The software was developed using a ResNet34-based deep convolutional neural network with 10 different abnormality-specific channels in the final layer. In clinical practice, it analyzes Postero-Anterior or Antero-Posterior projections of chest radiographs by identifying the following findings: atelectasis, calcification, cardiomegaly, consolidation, fibrosis, nodules, mediastinal widening, pleural effusion, pneumoperitoneum, and pneumothorax. As a result of the analysis, the device enables the visualization of the findings on the CXR and returns the interpretation of the abnormality (Fig. **1**), providing a quantitative estimation of the likelihood, as a percentage.

### 2.3 Radiological Assessment

For each patient, a blinded double reading was conducted by two general radiologists with less than one year of experience (RAD1 and RAD2). The two radiologists independently assessed the presence of the same range of abnormalities identified by the AI-based tool (atelectasis, calcification, cardiomegaly, consolidation, fibrosis, nodules, mediastinal widening, pleural effusion, pneumoperitoneum and pneumothorax). If the finding was considered uncertain, all alternative interpretations were also annotated among the abnormalities. The final database included every single finding identified by either the radiologists or the software. If a latero-lateral projection was available, the readers were allowed to use it.

Furthermore, after three months, the two radiologists reviewed all the examinations with the assistance of the software, confirming or denying the reported findings.

The gold standard was established by the senior radiologist (>10 years of experience in thoracic imaging), who assessed all the images, assisted by the software in the same way.

### 2.4 Statistical Analysis

Continuous variables were expressed as the mean and standard deviation. Categorical variables were expressed as counts and percentages. Sensitivity and specificity were computed for the detection of all the findings considered together and for each abnormality, compared to the gold standard. Chi-squared tests were used to compare the performances, with a significance threshold set at a two-sided 5% (*p* < 0.05). All statistical analyses were performed with SPSS (version 25.0, IBM Corp).

## 3 RESULTS

All first 500 CXRs were included, and the final dataset consisted of 300 males (60%), with a mean age of 55 years ± 1.5 SD. A total of 600 findings were reported by the senior radiologist. All CXR findings are shown in Table **1**. Pneumoperitoneum was never detected.

Considering all findings, RAD1 had a sensitivity of 80.6% and a specificity of 93.2%, RAD2 had a sensitivity of 82.9% and a specificity of 95.5%, and AI had a sensitivity of 96.9% and a specificity of 63.9%.

The sensitivity of RAD1 and RAD2 was significantly lower than that of AI (*p* < 0.001), while the specificity was significantly higher for both RADs (*p* < 0.001).

In particular, the sensitivity of RAD1 was significantly lower than that of AI for the detection of atelectasis (*p* = 0.042), consolidation (*p* < 0.001), fibrosis (*p* = 0.005), and nodules (*p* = 0.002). The sensitivity of RAD2 was significantly lower than that of AI for the detection of calcification (*p* < 0.001), fibrosis (*p* < 0.001), and pleural effusion (*p* = 0.042) (Fig. **2**).

The specificity of RAD1 was significantly higher than that of AI for the detection of atelectasis (*p* = 0.047), calcification (*p* < 0.001), consolidation (*p* < 0.001), fibrosis (*p* < 0.001), nodules (*p* < 0.001), pleural effusion (*p* = 0.004), and pneumothorax (*p* = 0.002) (Fig. **3**).

The specificity of RAD2 was significantly higher than that of AI for the detection of calcification (*p* < 0.001), consolidation (*p* = 0.001), fibrosis (*p* < 0.001), nodules (*p* < 0.001), and pneumothorax (*p* = 0.002) (all data shown in Table **2**.

Overall sensitivity increased in both RADs (*p* < 0.001) with the assistance of AI, while specificity decreased in both RAD1 (*p* = 0.004) and RAD2 (*p* < 0.001) with the assistance of AI.

Considering each finding, the sensitivity of RAD1 significantly increased with AI assistance for the detection of atelectasis (*p* = 0.002), consolidation (*p* < 0.001), fibrosis (*p* = 0.005), and nodules (*p* < 0.001). On the other hand, the sensitivity of RAD2 significantly increased with AI assistance for the detection of atelectasis (*p* = 0.013), calcification (*p* = 0.033), fibrosis (*p* = 0.030), and pleural effusion (*p* = 0.016).

The specificity of RAD1 significantly decreased with AI assistance for the detection of calcification (*p* = 0.001), consolidation (*p* < 0.001), fibrosis (*p* < 0.001), nodules (*p* < 0.001), pleural effusion (*p* = 0.012), and pneumothorax (*p* = 0.005). On the other hand, the specificity of RAD2 significantly decreased with AI assistance for the detection of fibrosis (*p* = 0.048) and pneumothorax (*p* = 0.014) (Table **3**).

Moreover, the sensitivity and specificity of RAD1 and RAD2 without AI assistance were similar when taking into account all abnormalities (p ≥ 0.127), but sensitivity was different in the detection of calcification (*p* < 0.001), cardiomegaly (*p* = 0.021), and consolidation (*p* < 0.001), while specificity was different for cardiomegaly (*p* < 0.001), consolidation (*p* < 0.001), mediastinal widening (*p* = 0.025), and pleural effusion (*p* = 0.034). With AI assistance, the two radiologists reached similar sensitivity for the detection of cardiomegaly and consolidation, and similar specificity for the detection of consolidation, mediastinal widening, and pleural effusion (Table **4**).

## 4 DISCUSSION

In this work, we verified the sensitivity and specificity of two general radiologists with less than one year of experience and of an AI-based software, and whether the performance of the readers may increase with the use of the computer-aided detection tool compared to that of an experienced radiologist [13-15].

Considering the total findings, the readers exhibited lower sensitivity compared to AI but higher specificity; this result aligns with other available studies that included an expert radiologist as a reference standard. Ahn *et al*. [13] found similar results in 497 patients performing CXR using a similar version of the same software, which identified four findings: consolidation, pneumothorax, nodules, and pleural effusion. Kuo *et al*. [14] validated the performance of an AI model in detecting COVID-19-related abnormalities on CXR in 852 symptomatic patients. Lastly, Kvak *et al*. [15], in a study of 300 CXRs, verified an AI software capable of identifying 13 abnormalities, with additional findings compared to our software: pulmonary edema, bone fracture, hilar enlargement, and subcutaneous emphysema. All these studies revealed higher sensitivity and lower specificity for AI compared to radiologists relative to an expert reader.

Moreover, a recent study conducted by Plesner *et al*. [16] evaluated the diagnostic reliability of four commercially available AI tools for detecting airspace disease, pneumothorax, and pleural effusion on CXR in 2040 patients. Their results endorsed the notion that the current generation of AI systems demonstrates high sensitivity and excellent negative predictive values. However, the positive predictive values of these AI tools were notably lower. Hence, it clearly emerges that AI software achieves very high sensitivity, minimizing the number of false negatives as much as possible. Nonetheless, there is a non-negligible risk of overdiagnosis.

Our evaluation of the AI software as a second reader indicated an increase in sensitivity following the use of AI by the readers. This result partially aligns with findings from similar studies, in which all cases showed sensitivity improvements regardless of the radiologists' experience [5, 7, 17-19]. However, our study's divergence lies in the significant reduction of specificity observed with AI assistance, contrary to previous studies in which no significant variations in specificity were noted when readings were aided by AI [5, 7, 17-19]. The primary distinction in our study arises from the use of CXR as the reference standard, while the others relied on CT. Furthermore, in our work, image analysis was conducted with two radiologists with less than one year of experience, while other studies utilized more experienced radiologists.

A critical consideration is the significant reduction in specificity when using AI support, raising concerns about overdiagnosis that may lead to unnecessary follow-up investigations, increased patient anxiety, and additional healthcare costs. In particular, false positive findings while improving sensitivity could impair clinical decision-making if not carefully scrutinized by radiologists.

In our opinion, two possible interpretations may be raised: young radiologists could be influenced by AI outputs, or, conversely, experienced radiologists could be overly skeptical of AI results. Unfortunately, we cannot ascertain which hypothesis aligns with our results, thus necessitating further comparative research against CT. Moreover, the retrospective and single-center design inherently limits the generalizability of our findings. The exclusive use of two radiologists with limited experience may not reflect the behavior or performance of more seasoned practitioners, particularly in different healthcare settings. An in-depth analysis of discordant cases revealed that some false positives suggested by AI were accepted without critical review by the young readers, indicating a tendency toward an overreliance on AI when faced with uncertainty.

However, our results confirm that AI software serves as a valuable aid for radiologists as a second reader. By ensuring exceptional sensitivity, it should facilitate the detection of findings, enabling the radiologist to confirm or refute what has been highlighted by the software. This leads us to advocate for a balanced and critical approach when incorporating AI in radiology.

## 5 STUDY LIMITATIONS

There are several limitations in our study. Firstly, it was conducted retrospectively. Secondly, the reference standard was based on assessments made by a senior radiologist; while the current gold standard for lung imaging is CT, CXR is frequently employed in daily practice and may not always utilize CT for confirmatory purposes. Hence, understanding whether AI tools can improve the performance of less experienced radiologists in comparison to more trained readers is a critical research question. Lastly, the readers were permitted to allocate time to the task without constraints, which allowed for a focused examination of the images. This may overestimate the actual diagnostic performance relative to a real-world clinical setting, where time pressure, multitasking, and limited resources frequently impact radiological decisions.

## CONCLUSION

This study demonstrates that AI-assisted interpretation of chest X-rays can significantly improve the sensitivity of young radiologists in detecting thoracic abnormalities, albeit at the cost of reduced specificity. Despite this trade-off, the use of AI tools such as Lunit INSIGHT CXR can serve as a valuable second reader, particularly for less experienced practitioners. By enhancing diagnostic confidence and minimizing missed findings, AI holds promise as a supportive technology in routine radiological practice provided it is used with critical judgment and in complement to human expertise.

## Figures and Tables

**Fig. (1):**
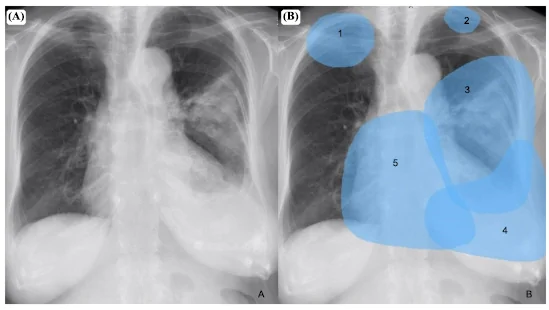
Postero-anterior orthostatic CRX in a 58-year-old female (**A**). AI-based software identified five findings (**B**): fibrosis (1 and 2), consolidation (3), pleural effusion (4) and cardiomegaly (5), confirmed by the senior radiologist.

**Fig. (2):**
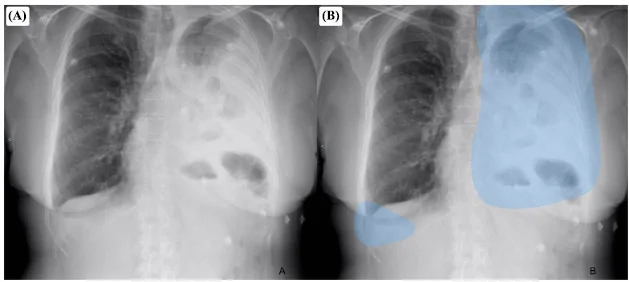
Postero-anterior orthostatic CRX in a 52-year-old female (**A**). AI-based software identified (**B**) the presence of extensive left pleural effusion, as the young and senior radiologists. It also highlights a slight basal pleural effusion on the right, a finding not identified by one reader (RAD2), but confirmed by the senior radiologist.

**Fig. (3):**
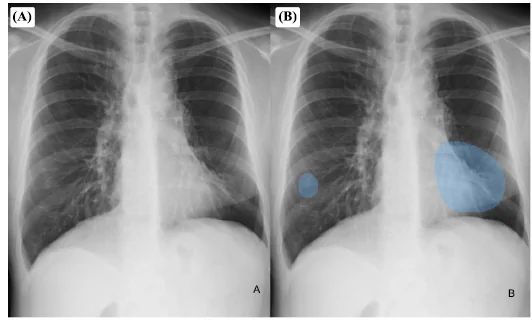
Postero-anterior orthostatic CRX in a 45-year-old male (**A**). AI-based software identified (**B**) the presence of a nodule on the right and consolidation on the left, not detected by the readers and not confirmed by the senior radiologist.

**Table 1:** CXR abnormalities by all readers.

CXR Abnormality	SENIOR RAD	RAD 1	RAD2	AI	RAD1+AI	RAD2 + AI
All findings	600	406	452	730	449	502
Atelectasis	70 (11,6%)	62 (15,2%)	89 (19,6%)	87 (11,9%)	62 (13,8%)	91 (18,1%)
Calcification	33 (5,5%)	23 (5,6%)	10 (2,2%)	48 (6,5%)	34 (7,5%)	17 (3,3%)
Cardiomegaly	56 (9,3%)	52 (12,8%)	35 (7,7%)	43 (5,8%)	52 (11,5%)	35 (6,9%)
Consolidation	157 (26,1%)	91 (22,4%)	147 (32,5%)	171 (23,4%)	94 (20,9%)	152 (30,2%)
Fibrosis	61 (10,1%)	27 (6,6%)	24 (5,3%)	91 (12,4%)	41 (9,1%)	35 (6,9%)
Nodule	92 (15,3%)	52 (12,8%)	54 (11,9%)	173 (23,6%)	66 (14,6%)	65 (12,9%)
Mediastinal w.	15 (2,5%)	14 (3,4%)	6 (1,3%)	8 (1%)	14 (3,1%)	7 (1,3%)
Pleural effusion	90 (15%)	70 (17,2%)	73 (16,1%)	83 (11,3%)	71 (15,8%)	78 (15,5%)
Pneumothorax	26 (4,3%)	15 (3,6%)	14 (3%)	26 (3,5%)	15 (3,3%)	22 (4,3%)

**Abbreviations:** AI: artificial intelligence based software. CXR: chest X-ray. Mediastinal w.: mediastinal widening. RAD1: radiologist 1. RAD2: radiologist 2.

**Table 2:** Sensitivity and specificity of radiologists and AI.

CXR abnormality	RAD1		RAD2		AI		RAD1 *vs* AI (*p*-value)		RAD2 *vs* AI (*p*-value)	
	SENS	SPEC	SENS	SPEC	SENS	SPEC	SENS	SPEC	SENS	SPEC
All findings	80.6%	93.2%	82.9%	95.5%	96.9%	63.9%	<0.001*	<0.001*	<0.001*	<0.001*
Atelectasis	70.2%	97.3%	78.9%	95.7%	86.0%	95.5%	0.042*	0.047*	0.325	0.809
Calcification	93.8%	99.1%	25.0%	99.4%	87.5%	96.0%	0.544	<0.001*	<0.001*	<0.001*
Cardiomegaly	100%	98.3%	86.8%	98.9%	97.4%	99.3%	0.314	0.072	0.089	0.058
Consolidation	68.5%	99.5%	92.9%	96.8%	92.9%	93.2%	<0.001*	<0.001*	1.000	0.001*
Fibrosis	69.0%	99.2%	48.3%	99.3%	96.6%	92.6%	0.005*	<0.001*	<0.001*	<0.001*
Nodule	61.5%	95.8%	84.6%	96.5%	96.2%	82.8%	0.002*	<0.001*	0.158	<0.001*
Mediastinal widening	100%	99.4%	66.7%	100%	77.8%	99.9%	0.134	0.102	0.599	0.317
Pleural effusion	93.2%	99.8%	86.3%	99.9%	95.9%	98.4%	0.467	0.004*	0.042*	0.390
Pneumothorax	93.8%	100%	87.5%	100%	100%	98.8%	0.309	0.002*	0.144	0.002*

**Abbreviations:** AI: artificial intelligence based software. CXR: chest X-ray. SENS: sensitivity. SPEC: specificity.

**Note:** *Statistically significant. RAD1: radiologist 1. RAD2: radiologist 2.

**Table 3:** Sensitivity and specificity of radiologists without and with AI-assistance.

CXR abnormality	RAD1		RAD 1+ AI		RAD*1vs* RAD1+AI (*p-*value)		RAD2		RAD2+AI		RAD2 *vs* RAD2+AI (*p-*value)		
	SENS	SPEC	SENS	SPEC	SENS	SPEC	SENS	SPEC	SENS	SPEC	SENS		SPEC
All findings	80.6%	93.2%	99.7%	87.8%	<0.001*	0.004*	82.9%	95.5%	99.0%	89.5%	<0.001*		<0.001*
Atelectasis	70.2%	97.3%	93.0%	96.2%	0.002*	0.209	78.9%	95.7%	94.7%	95.6%	0.013*		0.906
Calcification	93.8%	99.1%	93.8%	96.9%	1.000	0.001*	25.0%	99.4%	62.5%	99.0%	0.033*		0.283
Cardiomegaly	100%	98.3%	100%	98.8%	1.000	0.411	86.8%	99.9%	92.1%	99.9%	0.455		1.000
Consolidation	68.5%	99.5%	97.6%	94.3%	<0.001*	<0.001*	92.9%	96.8%	96.9%	96.3%	0.155		0.572
Fibrosis	69.0%	99.2%	96.6%	93.9%	0.005*	<0.001*	48.3%	99.3%	75.9%	98.2%	0.030*		0.048*
Nodule	61.5%	95.8%	100%	85.4%	<0.001*	<0.001*	84.6%	96.5%	88.5%	95.3%	0.685		0.222
Mediastinal w.	100%	99.4%	100%	99.9%	1.000	0.102	66.7%	100%	88.9%	99.8%	0.257		0.563
Pleural effusion	93.2%	99.8%	98.6%	98.6%	0.095	0.012*	86.3%	98.9%	97.3%	99.3%	0.016*		0.436
Pneumothorax	93.8%	100%	93.8%	99.1%	1.000	0.005*	87.5%	100%	100%	99.3%	0.144		0.014*

**Abbreviations:** AI: artificial intelligence based software. CXR: chest X-ray. Mediastinal w: mediastinal widening. SENS: sensitivity. SPEC: specificity.

**Note:** *Statistically significant (p<0.05). RAD1: radiologist 1. RAD2: radiologist 2.

**Table 4:** Comparison of sensitivity and specificity of radiologists with and without AI-assistance.

CXR abnormality	RAD1 *vs* RAD2 (*p*-value)		RAD1+AI *vs* RAD2+AI (*p*-value)	
	SENS	SPEC	SENS	SPEC
All findings	0.405	0.127	0.178	0.418
Atelectasis	0.282	0.080	0.697	0.532
Calcification	<0.001*	0.404	0.033*	0.002*
Cardiomegaly	0.021*	<0.001*	0.077	0.007*
Consolidation	<0.001*	<0.001*	0.702	0.068
Fibrosis	0.110	0.783	0.022*	<0.001*
Nodule	0.061	0.451	0.074	<0.001*
Mediastinal w.	0.059	0.025*	0.304	0.563
Pleural effusion	0.173	0.034*	0.560	0.223
Pneumothorax	0.544	1.000	0.310	0.592

**Abbreviations:** AI: artificial intelligence based software. CXR: chest X-ray. Mediastinal w: mediastinal widening. SENS: sensitivity. SPEC: specificity.

**Note:** *Statistically significant (p<0.05). RAD1: radiologist 1. RAD2: radiologist 2.

## Data Availability

All data generated or analyzed during this study are included in this published article.

## LIST OFABBREVIATIONS

AI: Artificial Intelligence
CXR: Chest X-ray
CT: Computed Tomography
RAD: Radiologist

## STANDARDS OF REPORTING

STROBE guidelines were followed.
